# Data for efficiency comparison of raw pumice and manganese-modified pumice for removal phenol from aqueous environments—Application of response surface methodology

**DOI:** 10.1016/j.dib.2018.09.027

**Published:** 2018-09-14

**Authors:** Maryam Heydari, Kamaladdin Karimyan, Mohammad Darvishmotevalli, Amir Karami, Yasser Vasseghian, Nahid Azizi, Mehdi Ghayebzadeh, Masoud Moradi

**Affiliations:** a Department of Environmental Health Engineering, Faculty of Public Health, Tehran University of Medical Sciences, Tehran, Iran; b Environmental Health Research Center, Kurdistan University of Medical Sciences, Sanandaj, Iran; c Environment Research Center, Isfahan University of Medical Sciences, Isfahan, Iran; d Research Center for Environmental Determinants of Health, Kermanshah University of Medical Sciences, Kermanshah, Iran; e Students Research Committee, Kermanshah University of Medical Sciences, Kermanshah, Iran; f Health and Environment Research Center, Department of Environmental Health Engineering, Tabriz University of Medical Sciences, Tabriz, Iran

**Keywords:** Phenol, Manganese-modified pumice, RSM, Aqueous environment

## Abstract

Present deadest collection was aimed to evaluate the efficiency of raw pumice (RWP) and Mn-modified pumice (MMP). Response surface methodology (RSM) based on the central composite designs (CCD) was applied to evaluate the effects of independent variables including pH, adsorbents dosage, contact time and adsorbate concentration on the response function and the best response values were predicted. The Fourier transform infrared spectroscopy (FTIR), X-ray diffraction (XRD) and scanning electron microscopy (SEM) were used to characterize the adsorbents. Based on acquired data, the maximum efficiency removal of phenol was obtained 89.14% and 100% for raw and Mn-modified pumice respectively. The obtained data showed pH was effective parameter on phenol removal among the different variables. Evaluation of data using isotherms and kinetics models showed the fitted with Langmuir isotherm and pseudo second order kinetic for both adsorbents. According to obtained data was observed that modification of pumice can improve the efficiency removal of phenol to meet the effluent standards.

**Specifications table**

**Table:** 

Subject area	Environmental Health Engineering
More specific subject area	Environmental Chemistry
Type of data	Tables, figures, text file
How data was acquired	The performance of RWP and MMP were evaluated to removing of phenol from aqueous solution. The characteristics of adsorbents were conducted by SEM, XRD and FTIR analysis. The response surface methodology (RSM) was used for analyzing the effects of several independent variables (pH, adsorbate concentration, contact time and adsorbents dosage) on the response. Moreover, obtained data were evaluated by isotherms and kinetics equations.
Data format	Raw, analyzed
Experimental factors	All samples were kept in polyethylene bottles in a dark place at room temperature.
Experimental features	The all above mentioned parameters were analyzed according to the standard method for water and wastewater treatment handbook [1].
Data source location	Kermanshah city, Iran
Data accessibility	Data are included in this article
Related research article	M. Moradi, A.M. Mansouri, N. Azizi, J. Amini, K. Karimi, K. Sharafi, Adsorptive removal of phenol from aqueous solutions by copper (Cu)-modified scoria powder: process modeling and kinetic evaluation, Desalin Water Treat. 57 (2016)11820–11834 [2].

**Value of the data**

• The obtained data of this dataset showed that Mn-modification effect on adsorbent led to increasing of equilibrium sorption capacity for removal of phenol.
• Due to cheap and high availability of this type of adsorbent in Iran, the efficiency of it can be improved by making these simple modifications and so the application of it in water and wastewater treatment will be increased.
• The obtained data of present dataset can be used for design and development of future similar studies. Because in this study, the optimal conditions for the removal of phenol by FSP are determined. Therefore, the range of future study variables can be determined based on the optimal conditions of this dataset.
• The raw data of this dataset was analyzed using the RSM method [3], [4], [5], [6]. Therefore, the data related to the optimization conditions and the determination of the effect of each parameter will be very understandable for other researchers.

## 1 Data

Table 1 shows the experimental conditions and results of central composite design. The obtained data indicated the maximum efficiency removal of phenol was obtained 89.14% and 100% for RWP and MMP respectively. Table 2, Table 3 revealed the estimated regression coefficients and ANOVA dataset from the central composite design experiments for RWP and MMP respectively.

**Table 1:** Experimental conditions and results of central composite design.

**Run**	**Variables**				**Response (Phenol removal by RWP)**		**Response (Phenol removal by MMP)**	
	**Factor 1**	**Factor 2**	**Factor 3**	**Factor 4**	**Actual**	**Predicted**	**Actual**	**Predicted**
	**A: pumice dosage (g/l)**	**B: Contact time (min)**	**C: pH**	**D: Phenol concentration (mg/l)**	**%**	**%**	**%**	**%**
1	1	20	11	50	19.31	18.97	29.6	27.87
2	0.2	20	11	50	6.21	6.9	11.7	12.31
3	1	20	3	50	79.68	81.46	89.64	91.7
4	0.6	80	7	150	70.52	67.65	78.92	73.91
5	1	100	11	50	29.32	27.89	34.55	32.23
6	0.6	60	7	150	65.76	65.42	71.98	73.28
7	1	100	3	250	68.61	65.04	76.46	75.28
8	0.6	60	7	150	65.76	65.42	72.28	73.28
9	0.6	60	7	100	66.27	69.58	76.87	76.72
10	0.2	100	3	250	49.84	52.97	61.25	63.6
11	0.6	40	7	150	58.57	63.19	65.86	70.1
12	0.6	60	7	150	65.76	65.42	72.67	73.28
13	0.6	60	7	200	60.73	61.26	69.41	68.79
14	0.6	60	7	150	65.76	65.42	72.67	73.28
15	0.6	60	7	150	65.76	65.42	72.67	73.28
16	0.2	100	11	50	14.17	15.82	11.7	15.73
17	0.6	60	9	150	53.06	48.87	58.97	56.18
18	1	100	3	50	89.14	90.38	100	102.85
19	0.2	20	11	250	3.94	-1.03	10.36	8.12
20	0.4	60	7	150	57.44	59.23	64.82	67.94
21	0.8	60	7	150	69.07	65.26	78.64	74.75
22	0.6	60	5	150	73.6	75.76	82.96	84.98
23	0.2	100	11	250	8.53	7.89	14.88	12.25
24	1	100	11	250	15.19	19.96	20.64	23.36
25	1	20	3	250	59.04	56.12	66.85	63.44
26	1	20	11	250	8.93	11.04	15.44	18.3
27	0.2	20	3	250	41.01	44.05	50.95	52.7
28	0.2	20	3	50	70.38	69.38	77.68	75.58
29	0.2	100	3	50	81.34	78.31	89.21	85.78
30	0.6	60	7	150	65.76	65.42	74.5	73.28

**Table 2:** Estimated regression coefficients and corresponding to ANOVA results from the data of central composite design experiments before elimination of insignificant model terms: (RWP).

**MT**	**CE**	**SE**	**SS**	**DF**	**MS**	**FV**	**PV**	**S/NS**
Quadratic model	–	–	18,744.97	14	1338.93	128.20	< 0.0001	Significant
A	6.04	0.80	601.40	1	601.40	57.58	< 0.0001	Significant
B	4.46	0.80	328.43	1	328.43	31.45	< 0.0001	Significant
C	−26.89	0.80	11,932.03	1	11,932.03	1142.47	< 0.0001	Significant
D	−8.32	0.80	1141.34	1	1141.34	109.28	< 0.0001	Significant
AB	0.18	0.81	0.55	1	0.55	0.052	0.8220	Not significant
AC	−0.88	0.81	12.25	1	12.25	1.17	0.2959	Not significant
AD	0.19	0.81	0.60	1	0.60	0.058	0.8137	Not significant
BC	−0.62	0.81	6.25	1	6.25	0.60	0.4512	Not significant
BD	−0.57	0.81	5.22	1	5.22	0.50	0.4904	Not significant
CD	4.35	0.81	302.93	1	302.93	29.01	< 0.0001	Significant
A^2^	−7.91	7.92	10.40	1	10.40	1.00	0.3341	Not significant
B^2^	−2.75	7.92	1.25	1	1.25	0.12	0.7337	Not significant
C^2^	−7.61	7.92	9.63	1	9.63	0.92	0.3522	Not significant
D^2^	−6.93	7.92	7.98	1	7.98	0.76	0.3958	Significant

**CE:** Coefficient Estimate, **SE:** Standard Error, **MT:** Model Terms, **SS:** Sum of squares, **DE:** Degree of Freedom, **MS:** Mean square, **FV:** F-value, **PV:** P-value, **S:** Significant, **NS:** Not significant

**Table 3:** Estimated regression coefficients and corresponding to ANOVA results from the data of central composite design experiments before elimination of insignificant model terms: (MMP).

**MT**	**CE**	**SE**	**SS**	**DF**	**MS**	**FV**	**PV**	**S/NS**
Quadratic model	–	–	20,758.16	14	1482.73	118.25	< 0.0001	Significant
A	6.81	0.87	765.14	1	765.14	61.02	< 0.0001	Significant
B	3.82	0.87	240.55	1	240.55	19.18	0.0005	Significant
C	−28.80	0.87	13,683.74	1	13,683.74	1091.28	< 0.0001	Significant
D	−7.94	0.87	1039.74	1	1039.74	82.92	< 0.0001	Significant
AB	0.24	0.89	0.89	1	0.89	0.071	0.7937	Not significant
AC	−0.14	0.89	0.32	1	0.32	0.026	0.8748	Not significant
AD	−1.35	0.89	29.03	1	29.03	2.31	0.1490	Not significant
BC	−1.70	0.89	46.00	1	46.00	3.67	0.0747	Not significant
BD	0.17	0.89	0.49	1	0.49	0.039	0.8465	Not significant
CD	4.67	0.89	349.60	1	349.60	27.88	< 0.0001	Significant
A^2^	−7.73	8.68	9.95	1	9.95	0.79	0.3871	Not significant
B^2^	−5.09	8.68	4.32	1	4.32	0.34	0.5662	Not significant
C^2^	−10.79	8.68	19.38	1	19.38	1.55	0.2328	Not significant
D^2^	−2.09	8.68	0.73	1	0.73	0.058	0.8128	Significant

**CE:** Coefficient Estimate, **SE:** Standard Error, **MT:** Model Terms, **SS:** Sum of squares, **DE:** Degree of Freedom, **MS:** Mean square, **FV:** F-value, **PV:** P-value, **S:** Significant, **NS:** Not significant

**Fig. 1:**
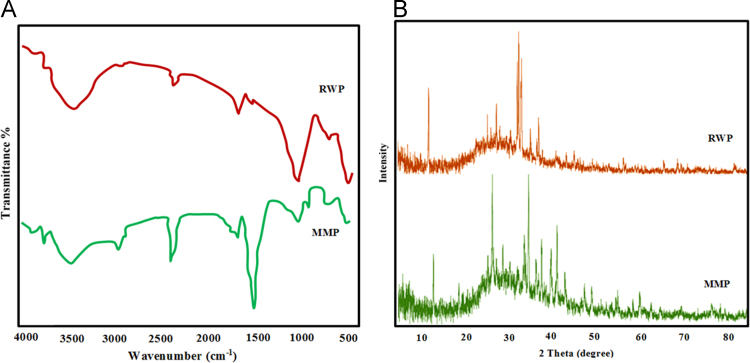
A) Fourier transform infrared spectroscopy (FTIR) and B) XRD patterns of RWP and MMP.

**Fig. 2:**
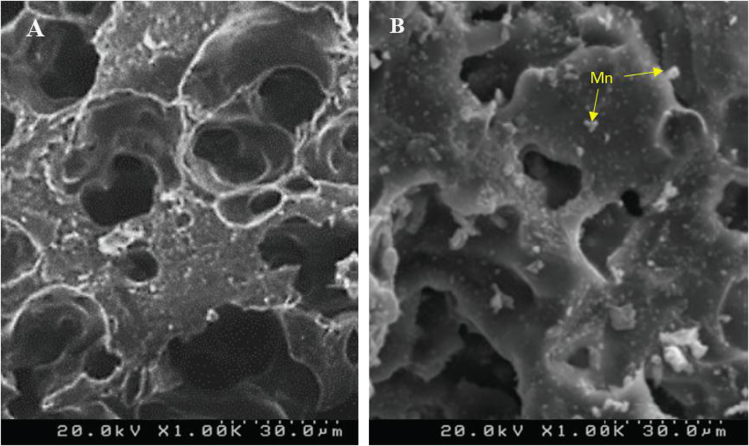
SEM images of A) RWP and B) MMP.

**Fig. 3:**
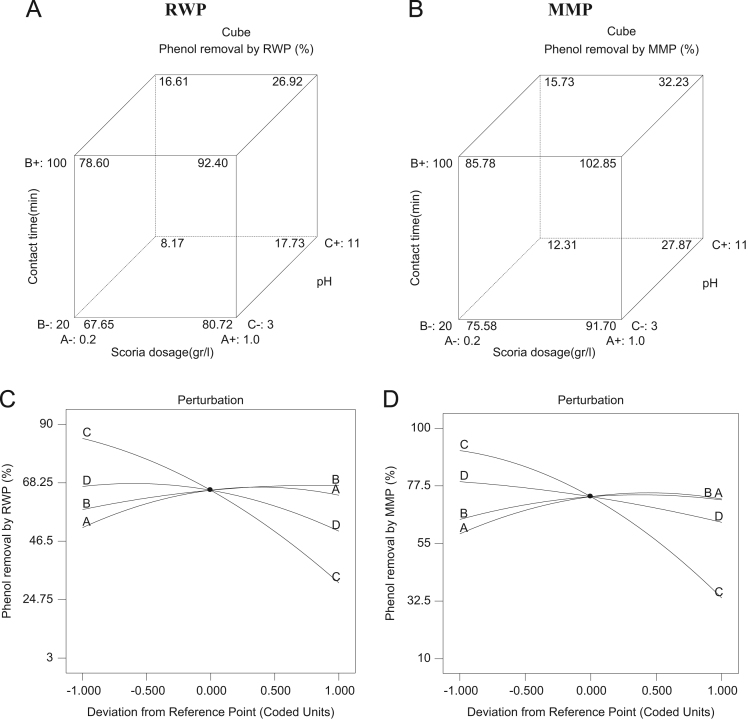
Trend of phenol removal efficiency by RWP and MMP with respect to pumice dosage (A), contact time (B), pH (C), and phenol concentration (D).

**Fig. 4:**
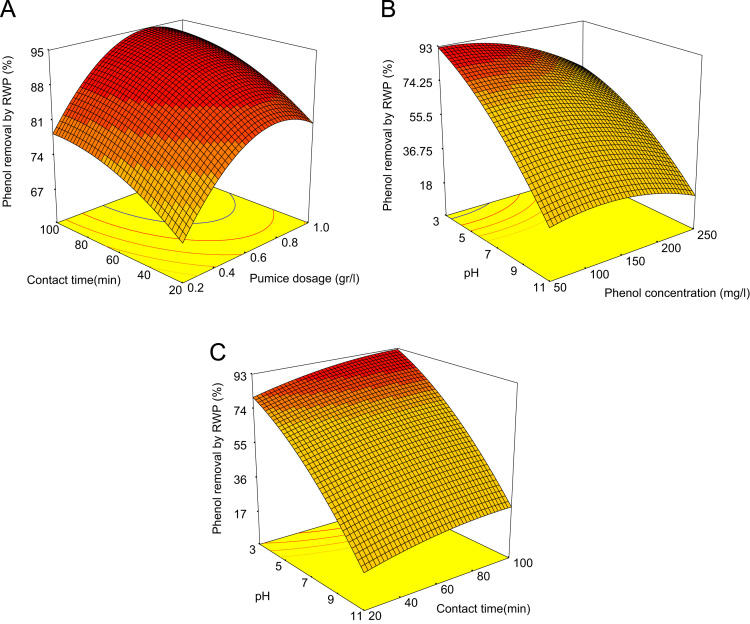
Response surface plots for phenol removal efficiency by RWP with respect to contact time and Pumice dosage (A), pH and phenol concentration (B), pH and contact time (C).

**Fig. 5:**
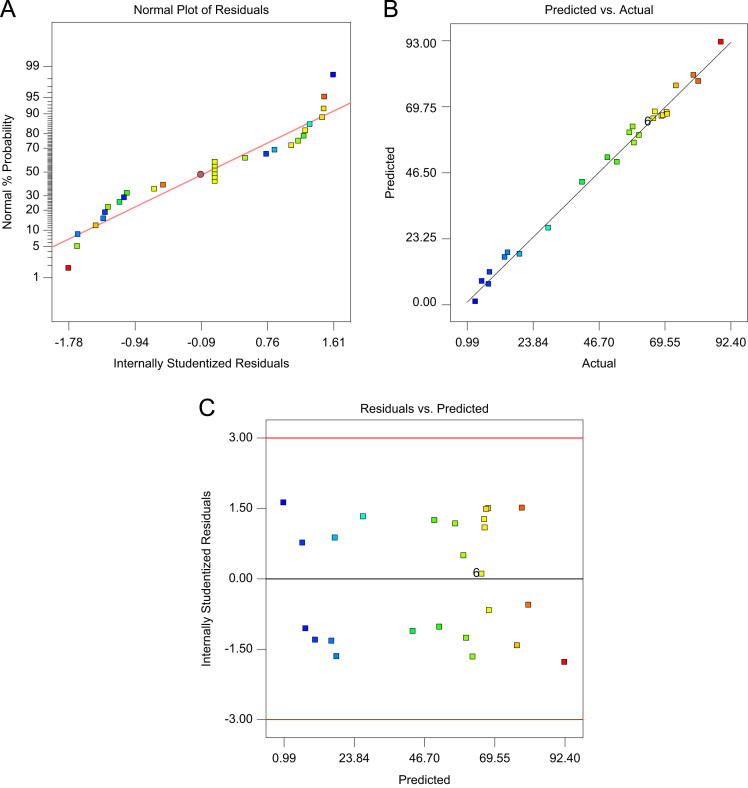
Normal probability plot of residual (A), predicted vs. actual values plot (B), and plot of residual vs. predicted response (C) related to phenol removal efficiency by RWP.

**Fig. 6:**
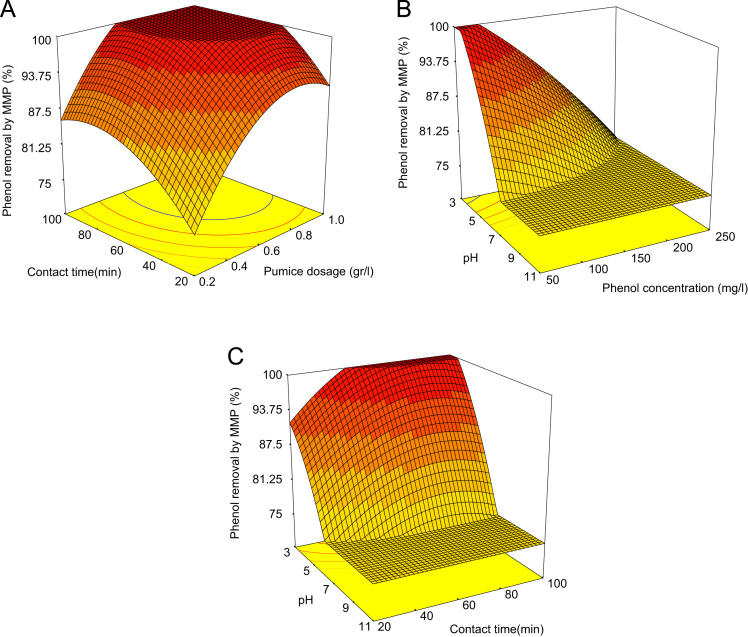
Response surface plots for phenol removal efficiency by MMP with respect to contact time and pumice dosage (A), pH and phenol concentration (B), pH and contact time (C).

**Fig. 7:**
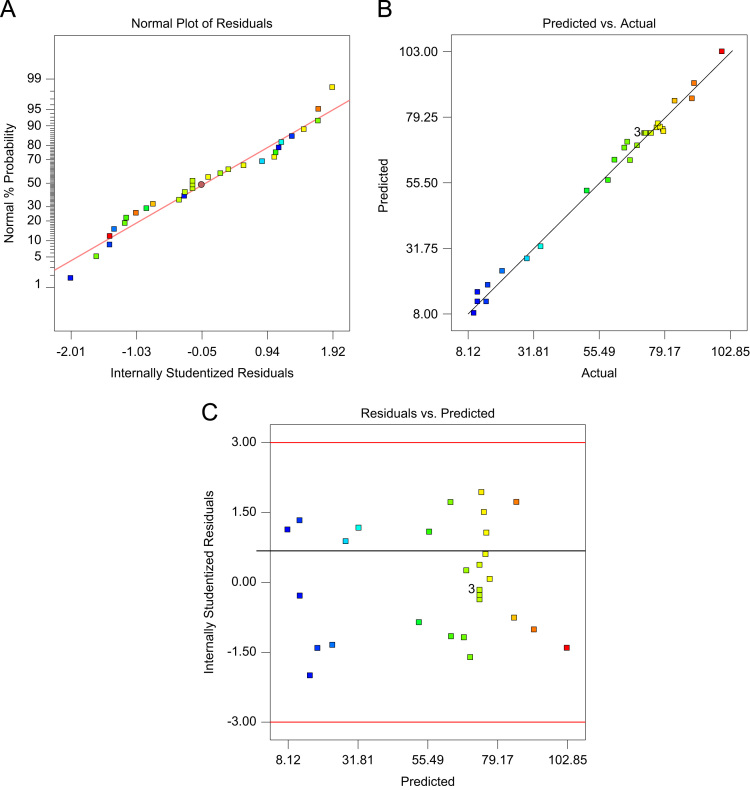
Normal probability plot of residual (A), predicted vs. actual values plot (B), and plot of residual vs. predicted response (C) related to phenol removal efficiency by MMP.

Table 4 indicated Analysis of variance (ANOVA) for fit of Phenol removal efficiency by RWP and MMP. Table 5 shows the parameters of Langmuir and Freundlich isotherms for phenol adsorption on RWP and MMP. The acquired data indicated the data were obeyed the Langmuir isotherm for RWP (R^2^=0.9798) and MMP (R^2^=0.9944). Also, Table 6 indicates kinetic model parameters. The revealed data were obey the pseudo second order for RWP (R^2^=0.9748) and MMP (R^2^=0.9971). Fig. 1 illustrates the Fourier transform infrared spectroscopy (FTIR) and XRD patterns of RWP and MMP. Fig. 2 demonstrates the SEM images of RWP and MMP. Fig. 3 shows trend of phenol removal efficiency by RWP. Fig. 4 shows the response surface plots for phenol removal efficiency by RWP. Fig. 5 indicated the normal probability plot of residual related to phenol removal efficiency by RWP. Fig. 6 shows the response surface plots for phenol removal efficiency by MMP. Fig. 7 indicated the normal probability plot of residual related to phenol removal efficiency by MMP.

**Table 4:** Analysis of variance (ANOVA) for fit of phenol removal efficiency by RWP and MMP from central composite design after elimination of insignificant model terms.

**Adsorbent**	**Model**	**SMT**	**SD**	**R**^**2**^	**Adj. R**^**2**^	**CV**	**AP**	**PRESS**	**PV**	**FV**	**PLF**
RWP	Quadratic	A,B,C,D, CD	3.23	0.991	0.984	6.26	40.03	1090.93	<0.0001	128.2	0.081
MMP	Quadratic	A,B,C,D, CD	3.54	0.991	0.982	5.99	37.83	1280.44	<0.0001	118.2	0.004

Removal phenol by RWP(%)= 6.04A+4.46B-26.89C-8.32D+4.35CD+65.47											

Removal phenol by MMP(%)=6.81A+3.82B-28.80C-7.94D+4.67CD+73.28											

**R**^**2**^**:** Determination Coefficient, **Adj. R**^**2**^**:** Adjusted R^2^, **AP:** Adequate Precision, **SMT:** Significant Model Terms, **SD:** Standard Deviation, **CV:** Coefficient Of Variation, **PRESS:** Predicted Residual Error Sum of Squares, **FV:** F-value, **PV:** P-value, **PLF:** Probability for Lack of Fit

**Table 5:** Isotherm equation parameters for phenol adsorption on RWP and MMP.

**Adsorbent**	**Langmuir isotherm**	
RWP	q_m_ (mg/g)	27.61
	b	0.4
	r^2^	0.9798
MMP	q_m_ (mg/g)	41.68
	b	0.095
	r^2^	0.9944
**Freundlich isotherm**		
RWP	n_T_	7.25
	K_f_ (mg/g(L/mg)^1/n^)	14.31
	r^2^	0.5332
MMP	n_T_	5.36
	K_f_ (mg/g(L/mg)^1/n^)	15.86
	r^2^	0.9078

**Table 6:** Kinetic model parameters for the adsorption phenol at different concentration on FSP.

**Kinetic model parameters**	**Kinetic parameters**	**Adsorbent type**	
		**RWP**	**MMP**
Pseudo-first-order	K_1_	0.227	0.206
	R^2^	0.9654	0.9852
Pseudo-second-order	K_1_	0.002	0.004
	R^2^	0.9948	0.9971
Pore diffusion	K_i_	1.03	0.9535
	R^2^	0.945	0.8968
Elovich	A	0.0973	0.22
	B	3.28	2.85
	R^2^	0.982	0.9705

## 2 Experimental design, materials and methods

### 2.1 Pumice preparation and its modification using manganese

Early preparations of raw scoria powder (RSP) were performed according to Moradi et al. study [2]. The coating of particles with manganese (Mn) was carried out as follows: 150 mL of 0.01 M Mn (NO_3_)_2_ solution and certain amount of raw pumice powder were transferred to a beaker. Then, pH was adjusted via HCl and NaOH 0.5 M. The beaker was putted on shaker at ambient temperature (25 °C ± 1 °C) for 72 h and dried at 105 °C for 24 h. the uncoated Mn was removed several times by distilled water and dried in 105 °C for 24 h [7].

### 2.2 Characteristics of RWP and MMP

The Fourier transform infrared spectroscopy (FTIR) analysis was conducted by WQF-510 Model. The chemical characteristics and surface morphology were determined by an XRD (Shimadzu XRD-6000) and scanning electron microscope (SEM; Philips XL30) respectively were used to Characteristics of RWP and MMP [8], [9], [10], [11].

### 2.3 Experimental design using RSM

Because the existence of many parameters which affected the results of experiments, achieve to the optimal conditions of experiments is an important strategy for determining the effective parameters and reducing the costs. Hence, attention to mathematical methods was developed to evaluate the obtained data. RSM based on central composite design (CCD) is a proper method to determine the best conditions of experiments for minimization of number of experiments and to survey of the relationship between the measured responses (phenol removal) and number of independent variables with the goal of optimizing the response [12], [13], [14], [15], [16]. (Design Expert 8.0, Stat-Ease Inc., Minneapolis, MN, USA) Table 7 illustrated- the experimental range and level of the independent variables.

**Table 7:** Experimental range and level of the independent variables.

**Variables**	**Range and level**				
	**−α(−1.5)**	**−1**	**0**	**1**	**+α(1.5)**
Contact Time, min	20	40	60	80	100
Adsorbent Dosage, g/l	0.2	0.4	0.6	0.8	1
pH	3	5	7	9	11
Phenol concentration, mg/l	50	100	150	200	250

### 2.4 Batch sorption studies

The sorption experiments were carried out in batch reactor. Initial concentration of phenol (50, 100, 150, 200 and 250 mg/l), adsorbent dose (0.1–1 g/L), pH (3, 5, 7, 9 and 11), contacted time (20, 40, 60, 80 and100 min) and ambient temperature (25 °C) were selected as variables. The residual phenol was determined by UV/VIS spectrophotometer (Hitachi Model 100-40) at λ_max_ 500 nm [17], [18], [19].

### 2.5 Adsorption isotherms and kinetics

The adsorbent capacity could be described using sorption isotherm. In the present study the adsorption data of phenol were evaluated by Langmuir and Freundlich isotherms. The linear Langmuir isotherm presented as follow:

(1) $$\frac{C_{e}}{q_{e}}=\frac{1}{bq_{m}}+\frac{C_{e}}{q_{m}}$$

Where the C_e_ is equilibrium concentration (mg/l), q_e_ is phenol adsorbed at equilibrium (mg/g), q_0_ and b are the Langmuir constants related to the capacity and energy of adsorption, respectively [20], [21].

Freundlich adsorption isotherm is an empirical expression that describes adsorption on a heterogeneous surface. The linear Freundlich isotherm could be illustrated as fallow:

(2) $$\ln q_{e}=\;\ln k_{f}\;+\;n^{-1}{\ln C}_{e}$$

Where K_f_ and n are Freundlich constants corresponded to adsorption capacity and adsorption intensity, respectively [22], [23], [24]. The kinetics were investigated via adsorption of certain concentration of phenol at different contact time. Kinetic study is essential for provide information on the factors affecting it reaction speed.

Several kinetics include pseudo-first-order, pseudo-second-order, intraparticle diffusion and elovich were used to controlling mechanisms of the adsorption process. The equations of kinetic models are expressed as follows [25], [26], [27], [28], [29], [30]:

Pseudo-first-order:

(3) $$\ln\left(q_{e}-q_{t}\right)=\;\ln q_{e}-k_{1}t$$

Pseudo-second-order:

(4) $$\frac{1}{q_{t}}=\frac{1}{q_{e}}+\;k_{2}t$$

Intraparticle diffusion:

(5) $$q_{t}\;=\;k_{p}t^{0.5}$$

Elovich:

(5) $$q_{t}\;=\;\beta \;\ln\left(\alpha \beta \right)\;+\;\beta \;\mathrm{lnt}$$
